# Waardenburg-Shah syndrome rare and challenging case report from Somalia

**DOI:** 10.1016/j.ijscr.2022.106952

**Published:** 2022-03-15

**Authors:** Abdishakur Mohamed Abdi, Abdullahi Yusuf Ali, Ismail Hakki Göl

**Affiliations:** Pediatric Surgery Department in Mogadishu Somali Turkish Training and Research Hospital, Mogadishu, Somalia

**Keywords:** Waardendenburg-Shah syndrome, Waardendenburg-Shah syndrome type 4, Intestinal agangliosis, Hirschsprung disease

## Abstract

**Introduction and importance:**

Waardenburg-Shah disorder could be an uncommon autosomal recessive inherited ailment characterized by aganglionic megacolon with a high mortality rate. Babies born with Waardenburg syndrome may have typical features of hair, skin and eye pigmentary abnormalities, and hearing loss. Here we present a case with typical presentation of Waardenburg Shah syndrome.

**Case presentation:**

This is a case of neonatal intestinal obstruction caused by a rare syndrome known as Waardenburg-Shah syndrome, with clinical manifestations of abdominal distension, bilious vomiting, and a history of delayed meconium passage with a family history of variant forms of this syndrome. The patients underwent first laparotomy, which found no atresia.post op colongarphy revealed a narrowed colon. Then reoperated, and a biopsy was taken and opened ileostomy. The pathology result showed gangilion negative. The patient was lost due to uncontrollable sepsis at the age of 2 months.

**Clinical discussion:**

Waardenurg syndrome is a congenital audito-pigmentary syndrome first described in 1951.Waardenurg syndrome is classified into four types, WS1 to WS4, and they share the common presence of congenital sensoneural hearing loss and pigmentary defects. The diagnosis of WS has major and minor criteria. The definitive management of this disorder involves surgical removal of aganglionic segment of the bowel and connecting functioning gangilioic bowel to the anus.

**Conclusion:**

Shah-Waardenburg syndrome TYPE-4 is a relatively unusual syndrome characterized by a higher prevalence of whole colonic aganglionosis with or without small bowel involvement, resulting in substantial morbidity and mortality in the neonatal age range.

## Highlights

• Waardenburg syndrome is a very rare genetically inherited disorder with a variety of manifestations characterized by agangilionic megacolon with high mortality rate with typical pigmentary abnormalities
• the definitive management of this disorder involves surgical removal of agangilionic segment of the bowel and connecting functioning gangilionic bowel to the anus
• These children's predicaments are exacerbated by inadequate health-care facilities. For better results, a comprehensive approach to these issues is required.

## 1 Introduction

Waardenburg syndrome is a very rare genetically inherited disorder with a variety of manifestations. Based on its presentation, which is divided into four categories: Types 1 through 4 are represented. Type 4 (Waardenburg Shah syndrome) [1]. The least common type Waardenburg Shah syndrome is characterized by sensory deafness, skin hair, and eye discoloration, as well as a broad nasal base, wade spaced epicanthi, and intestinal congenital aganglion (hrschsprung disease). Up to date, documented cases of Waardenburg Shah syndrome are limited. Wardenburg-Shah syndrome occurs in 1:50,000 live births [2]. We present the first case of Waardenburg Shah syndrome reported from Somalia, which presents to the hospital during the neonatal period with typical neonatal intestinal obstruction.

## 2 Case report

**Fig. 1:**
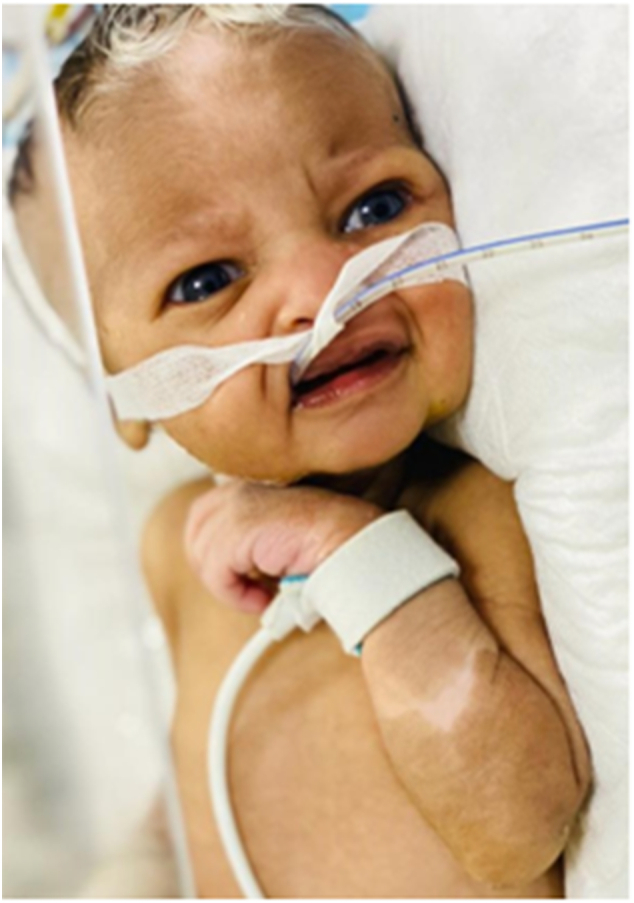
Photo showing hair, eye and skin discoloration.

**Fig. 2:**
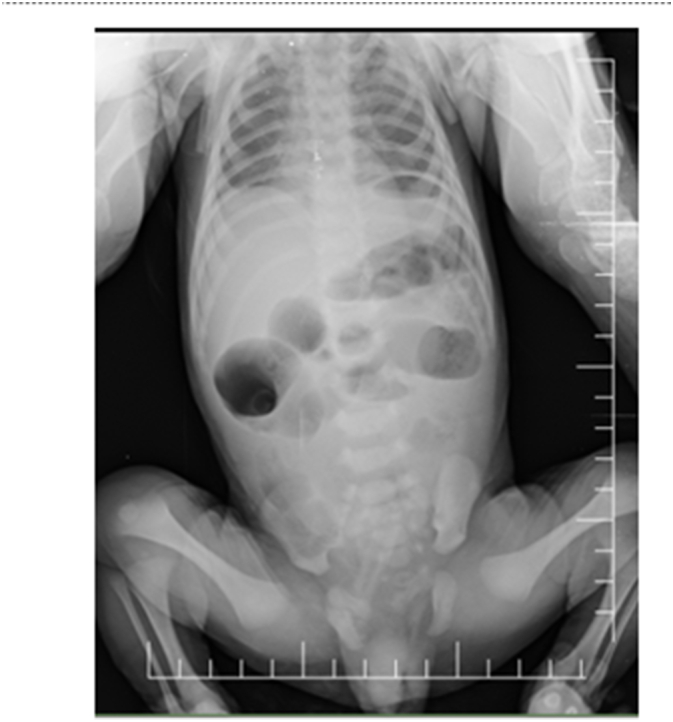
Erect abdominal X-ray with air fluid level.

**Fig. 3:**
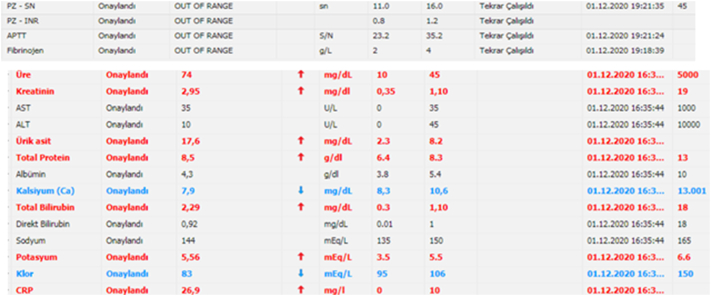
Abnormal laboratory parameters.

A 4-day-old full-term firstborn baby boy born to a 19-year-old mother was admitted to the neonatal ICU with complaints of bilious vomiting and delayed meconium passage. During clinical examination, we discovered forehead hair, eye, and right forearm discoloration. Fig. 1, and an erect abdominal X-ray revealed air fluid level, which is a radiological sign of intestinal obstruction, with suspected intestinal atresiawe as a periliminary diagnosis (Fig. 2). Blood analysis revealed elevated coagulation parameters, urea and creatinine (Fig. 3). Then started the correcting process: NG tube decompression and NPO.

After correcting the laboratory findings, we went to Operation, believing the cause of the intestinal obstruction was intestinal atresia. We made an incision of the right supra umbilical transverse incision and externated the whole intestine and searched for the atresia site but could not find it. Instead, we found a normal caliber of intestine to the terminal ileum, where intestinal caliber diminished. We controlled the patency of the lumen and it was patent. We thought this was caused by mild malrotation. Then we checked the large bowel and it looked normal after that closed abdomen.

For the following 8 days, we waited with the expectation of passing stool spontaneously, which unfortunately did not happen, and continued bilious drainage by NG tube. Then we did contrast clongraphy, which revealed a narrowed colon to the terminal ileum, which confirmed our suspicion of total colonic aganglionosis.

**Fig. 4:**
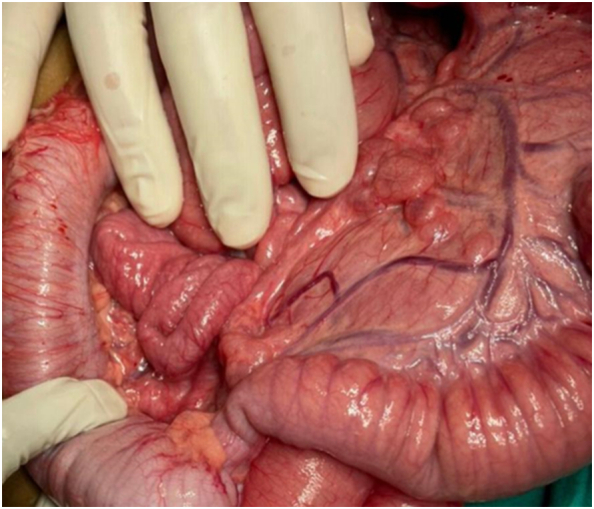
Intraoperative image of the aganglionic colon.

By searching for causes of total colonic hirschsprung disease with eye, hair, and skin discoloration, we came across the syndrome called WAARDENBURG SHAH SYNDEOME (TYPE IV). Later, we discovered through a family history that the patient's mother had a variant of Waardeburge syndrome with only right eye iris hypopigmentation and her grandmother had forelok discoloration, which indicates that this disease is genetically inherited. The following day, we operated on the neonate, taking a full-thickness biopsy from the terminal ileum to the rectosigmoid junction, opening a loop ileostomy 10 cm above the where-seems transition zone, and taking a biopsy from the stoma's mouth before closing the abdomen (Fig. 4). The pathology results revealed the presence of ganglion cells at the stoma site and an absence of ganglion cells from the terminal ileum to the rectum, which confirms total colonic Hirschsprung disease. Stoma started working on post-op day 3 and started oral feeding with a small amount and increased gradually until reaching full feeding regarding his age and being discharged. He was readmitted with.

He was readmitted with severe dehydration due to excessive stoma output and passed away at the age of 2 months due to intractable sepsis.

## 3 Discussion

Waardenurg syndrome is a congenital audito-pigmentary syndrome first described in 1951.

Waardenurg syndrome is classified into four types, WS1 to WS4, and they share the common presence of congenital sensoneural hearing loss and pigmentary defects. The diagnosis of WS has major and minor criteria. The following are the major criteria: congenital hearing loss, pigmentary disorders of the eyes and skin. Minor criteria include leukoderm hypopigmentation patches on the skin, a broad nasal bridge, incomplete nostril development, and premature gray hair before the age of 30.

For WS TYPE 1 to be diagnosed, two major criteria and one minor criteria must be met.WS TPE 2 is different from type 1 by the absence of dystopa canthorum (lateral displacement of the inner canthi). In addition to type-1 characteristics associated with musculoskeletal disorders (syndactyly, joint contractures, and muscle hypoplasia), WS TYPE-3This case did not show musculoskeletal abnormalities but had all the other features of Waardenburg syndrome. WS TYPE-4 is different from type-2 by the presence of Hirschsprung disease.

Babies born with Waardenburg syndrome may have typical features of hair, skin and eye pigmentary abnormalities and hearing loss, different manifestations of Waardenbug syndrome appear variably in different people [3]. Our case presented with all features except hearing loss. However, if the symptoms are mild, they may go undiagnosed. Some individuals may not need treatment or surgery, while others require both medical and surgical treatment. The identification of the syndrome has an important effect on the proper management of the patient. [4]. In our case, he underwent surgical intervention for purposes both diagnostic and therapeutic, by taking a biopsy from the intestine and opening an ilieostomy. R. Gupta reported a case of Waardenburge-Shah syndrome with total colonic agnagilionosis [5]. The current case belongs to Waadendengburg syndrome type-4 (Waardenbrg Shah syndrome) due to the presence of hirschsprungs disease associated with Waardenburg syndrome with total colon and distal ileum aganglionosis.

In type IV WS patients with EA features, postoperative consequences are similar to those reported in short bowel syndromes. These cases must deal with a variety of issues. Overgrowth of bacteria (sepsis, catheter blockage, liver damage and dysfunction) when it is still in its early stages these patients have a high mortality rate. Sepsis and hepatic failure are directly linked [6].

These children's predicaments are exacerbated by inadequate health-care facilities, a lack of knowledge, and illiteracy. For better results, a comprehensive approach to these issues is required. This study was reported in accordance with the SCARE 2020 guidelines [7].

## 4 Conclusion

Shah-Waardenburg syndrome TYPE-4 is a relatively unusual syndrome characterized by a higher prevalence of whole colonic aganglionosis with or without small bowel involvement, resulting in substantial morbidity and mortality in the neonatal age range. If these patients are not treated promptly, they will suffer crippling consequences. The use of preoperative frozen sections is critical in the proper care of such patients.

## Sources of funding

No funding was received.

## Ethical approval

Ethical approval was waived by the ethical committee of Mogadishu Somali Turkey, Recep Tayyip Erdogan Training and Research Hospital.

## Consent

Written informed consent was obtained from the patient for the publication of this case report and accompanying images. A copy of the written consent is available for review by the Editor-in-Chief of this journal on request.

## Author contribution

Abdishakur Mohamed Abdi. wrote the manuscript and corrected the manuscript for its scientific basis.

Abdullah Yusuf Ali. collected the data for the study.

Imail Hakki GÖl. director of the Department of Surgery and the consultant surgeon who provided the case

All authors have read and approved the final manuscript.

## Provenance and peer review

Not commissioned, externally peer-reviewed.

## Registration of research studies

N/a.

## Guarantor

Dr. Abdishakur Mohamed Abdi.

## Declaration of competing interest

This manuscript has not been submitted to, nor is it under review at, another journal or other publishing venue.

The authors have no affiliation with any organization with a direct or indirect financial interest in the subject matter discussed in the manuscript.
